# Effect of Short-Term Static Magnetic Field Pretreatment on Cold-Storage Quality and Phenolic Metabolism of Blueberries

**DOI:** 10.3390/foods15091505

**Published:** 2026-04-25

**Authors:** Ying Lu, Hui Liu, Zhenzhen Lv, Chengheng Li, Muhammad Nawaz, Qiang Zhang, Wenbo Yang, Jiechao Liu, Wenqiang Guan, Zhonggao Jiao

**Affiliations:** 1 Zhengzhou Fruit Research Institute, Chinese Academy of Agricultural Sciences, Zhengzhou 450009, China; 82101235560@caas.cn (Y.L.); lvzhenzhen@caas.cn (Z.L.); 82101235559@caas.cn (C.L.); mnawazafzal@outlook.com (M.N.); zhangqiang02@caas.cn (Q.Z.); yangwenbo@caas.cn (W.Y.); liujiechao@caas.cn (J.L.); 2 Zhongyuan Research Center, Chinese Academy of Agricultural Sciences, Xinxiang 453000, China; 3 College of Biotechnology and Food Science, Tianjin University of Commerce, Tianjin 300134, China; gwq18@163.com

**Keywords:** blueberry, static magnetic field, cold storage, polyphenol biosynthesis, antioxidant defense

## Abstract

Blueberries are widely consumed due to their richness in nutrients, yet they are also prone to quality deterioration after being harvested, even at cold temperatures. Non-thermal physical technology is an important auxiliary method worth considering for maintaining the quality of this fruit while refrigerated. In this study, a static magnetic field (SMF) was applied as a complementary pretreatment strategy prior to cold storage of blueberries. The optimal SMF parameters were identified as 5 mT exposure for 12 h, as this significantly retarded decay and softening. The contents of ascorbic acid, total polyphenols, flavonoids and proanthocyanidins were elevated by 20.0%, 17.7%, 23.9%, and 9.1%, respectively. Concurrently, DPPH (1,1-diphenyl-2-picrylhydrazyl) radical-scavenging capacity, catalase (CAT), and superoxide dismutase (SOD) activity markedly improved. Targeted metabolomic analysis revealed that SMF pretreatment significantly regulated polyphenol metabolic pathways and redirected polyphenol biosynthesis toward more stable and functional compounds, including three hydroxycinnamic acids, quercetin, dihydromyricetin, glycosylated hesperetin, and acylated delphinidin derivates. The synergistic effect of these SMF-elevated phenolics and the reinforced antioxidant system preserved the overall cold-storage quality of blueberries. These findings underscore the potential of SMF pretreatment as an effective physical technique for reducing postharvest blueberry losses.

## 1. Introduction

Blueberries (*Vaccinium corymbosum*) are widely cultivated and consumed due to their richness in nutritional compounds, including organic acids, iron, phenolic acids, flavonoids, anthocyanins, and ascorbic acid [1,2,3]. The 2024 Global Blueberry Industry Status Report published by the International Blueberry Organization (IBO) indicates continued growth in the blueberry industry, with total production reaching approximately 2.05 million tons. About 71.5% of blueberries are consumed fresh [4]. However, fresh blueberries are highly perishable after being harvested due to their rich nutrient content, high respiration rate, delicate epidermal layer, and high susceptibility to postharvest pathogenic diseases. The 20%~30% loss of postharvest blueberries significantly influences their economic value and market appeal [5]. With the increase in blueberry production scale, economic losses in the postharvest stage are becoming increasingly severe, highlighting the importance of developing safe and efficient preservation technologies.

Many highbush blueberry cultivars can maintain their marketable quality for approximately 2~4 weeks under conventional refrigerated storage (0~5 °C and high relative humidity). This storage time can only be extended with optimized assisted storage technologies or in specific cultivars [6,7]. Various assisted preservation techniques have been developed to improve lower-temperature storage and maintain the postharvest quality of blueberries, including modified atmosphere packaging (MAP) [8], controlled-atmosphere storage [6], ozone [9], ultraviolet-C (UV-C) [10], edible coatings [11], and natural antimicrobial agents [12]. These assisted methods effectively retard senescence and microbial spoilage, thereby extending cold-storage life. Physical techniques are generally safer, more environmentally friendly, and easier to implement, representing a promising direction for the preservation of postharvest fruit and vegetables. Magnetic field (MF) application, an advanced non-thermal physical method, has been proven to enhance the storage quality of various fruits and vegetables, including apples [13], peaches [13], strawberries [14], and cucumbers [15]. The preservation efficacy of MF-assisted technology is directly impacted by the type of MF [16], intensity [17], duration [18] and other parameters.

Continuous application of a static magnetic field over the entire cold storage period has been used in several previous studies [14,17,19]. However, the continuous operation of magnetic field devices for several weeks or months may impose practical limitations, such as an increased energy demand and moisture loss in fresh produce [20]. The utilization of SMFs as a short-term pretreatment method has received considerable attention recently, particularly in conjunction with precooling, offering a new perspective for the innovation of traditional precooling and preservation strategies. Zhao et al. [15] reported that combining an SMF with cold-water shock (70 Gs, 40 min) had a positive influence on cooling rates and improved catalase (CAT) and superoxide dismutase (SOD) activity, thereby maintaining the physicochemical properties of cucumbers during postharvest storage. A similar result was observed for magnetic-field-assisted hydrocooling pretreatment (4 mT, 30 min) with respect to cherry tomatoes, raising CAT activity and delaying postharvest ripening [16]. SMF pretreatment (3 mT, 30 min) before cold storage has also been found to effectively decrease the quantity of pectin-degrading enzymes in the cell wall and preserve sapodilla and wampee quality [18,21]. Although recent studies have indicated that SMF pretreatment, as a more energy-efficient approach, can improve the cold-storage quality of several fruits, the optimal SMF conditions for blueberry pretreatment remain underexplored.

Several potential mechanisms behind magnetic fields’ preservative effects on fresh fruit and vegetables have been identified, including decelerating microbial growth [22], suppressing fruit respiratory rate [23], maintaining energy states [23], increasing stress resistance [15], stabilizing cell membrane structure [24], and enhancing hydrogen bonding of water molecules, thereby reducing moisture loss [25]. Furthermore, magnetic field exposure has been reported to be able to regulate enzyme activity, since the Lorentz force from magnetic fields changes enzyme conformation via certain metal ions in active sites [15,26,27] and eliminates free radicals [28]. The preservation mechanism of SMFs, including as a pretreatment, is multifaceted and not yet fully understood.

The accumulation of endogenous reactive oxygen species (ROS) is generally correlated with cell membrane degradation and postharvest senescence. The antioxidant defense system plays a crucial role in eliminating ROS. Phenolics, as the major non-enzymatic antioxidants, could serve as a barrier against biotic and abiotic stress and are crucial for determining stress responsiveness, underpinning their essential contributions to antioxidant defense and postharvest longevity [29,30]. Understanding the impact of preservation technology on polyphenols is also important for improving health-related nutritional quality after harvest. Nevertheless, studies of the regulation of polyphenol metabolism in postharvest fruit pretreated via SMF are still lacking. The correlations between SMF-dependent phenolics and antioxidant defense and storage quality need to be revealed.

In this study, physiological, nutritional, and antioxidant attributes were systematically evaluated to optimize the intensity and exposure duration of short-term SMF pretreatment for blueberry storage. Subsequently, the regulation of blueberry phenolic biosynthesis by SMF pretreatment was investigated based on targeted polyphenol metabolomics. The results will help elucidate the role of magnetic field pretreatment in blueberry preservation, clarify its influence on metabolic changes, and provide practical insights for developing safe and sustainable postharvest technologies for reducing blueberry losses.

## 2. Materials and Methods

### 2.1. Sample Preparation, SMF Pretreatment and Cold Storage

Mature blueberries (*Vaccinium corymbosum* L. cv. Bluecrop) were harvested at a local commercial orchard in Lishui county (Pingdingshan, Henan Province, China) and immediately transported to our laboratory 3 h after being harvested. The initial soluble solid content and firmness of the fruit were 12.7 ± 0.84% and 1.83 ± 0.07 N, respectively, indicating a relatively uniform maturity level of the selected samples. Fruits with similar color, of uniform size (equatorial diameter: 1.2–1.5 cm; fruit weight, 1.5–2.0 g), and free from disease, pest damage, or mechanical injuries were selected for the experiment.

Blueberries were divided into five groups and subjected to different static magnetic field (SMF) intensities and exposure times, as shown below:

(1) CK: no SMF treatment (control);
(2) 5 mT-0.5 h: SMF pretreatment at 5 mT for 0.5 h;
(3) 5 mT-12 h: SMF pretreatment at 5 mT for 12 h;
(4) 10 mT-0.5 h: SMF pretreatment at 10 mT for 0.5 h;
(5) 10 mT-12 h: SMF pretreatment at 10 mT for 12 h.

The SMF treatment parameters were determined by comprehensively considering previous studies, preliminary experiments, and practical application scenarios. Specifically, 0.5 h was selected based on previously reported short-term magnetic field pretreatment conditions for postharvest fruits and vegetables [15,16,21,28] as well as its feasibility for rapid treatment during commercial postharvest handling of blueberries. We chose 12 h based on the time required for blueberries to be precooled to 0–1 °C in air so as to simulate a potential combined application of a magnetic field and postharvest precooling. The magnetic field intensities were selected based on the commonly used low-to-medium-intensity static magnetic field range reported in previous postharvest preservation studies [13,14,15,17]. The blueberries were placed in static magnetic field (SMF) refrigeration devices (MFF10, INDUC Scientific Co., Ltd., Wuxi, China) for pretreatment. A uniform constant magnetic field of 0~10 mT was generated by a power-supply-driven pair of square Helmholtz coils (80 cm × 80 cm; 400 turns) oriented vertically under excitation currents of 0~8 A.

After the SMF pretreatment, the blueberries were transferred into a temperature- and humidity-controlled cold-storage incubator (1 ± 0.5 °C, 90~95% RH; LHS-800HC, Wuxi Marit Technology Co., Ltd., Wuxi, China) and stored for 35 days. Sampling was performed on days 0, 7, 14, 21, 28 and 35 during storage.

All treatments were conducted with three biological replicates, with 2.5 kg of fruit in each replicate. Each 125 g portion of fruit was separately packed in a rigid plastic clamshell with the outer layer packaged in a perforated LDPE (low-density polyethylene, with a thickness of 0.02 mm) bag, which provided physical protection and allowed limited gas exchange during storage.

### 2.2. Firmness, Decay Percentage, and Color Change

Fruit firmness was determined using a texture analyzer (TA.XT Plus, Stable Micro Systems, Godalming, UK) equipped with a 2 mm diameter cylindrical probe. The test parameters were set as follows: pre-test speed, 3 mm/s; test speed, 2 mm/s; post-test speed, 3 mm/s; and compression distance, 7 mm. Ten blueberries were randomly selected per replicate, and each berry was measured twice at opposite equatorial positions. Firmness was expressed as the mean peak force (N) of measurements.

The blueberries were examined visually, and any fruit showing exudation, rot, or mycelial growth was classified as decayed fruit. The decay percentage (%) was calculated using Equation (1).

(1) $$Decay\;percentage\;(\%)=\frac{number\;of\;decayed\;fruit}{total\;number\;of\;fruit}\times 100$$

Chromatism (ΔE*) was used to evaluate the color change via a handheld spectrophotometer (CS-10, CHN Spec, Hangzhou, China). L*, a*, and b* values were recorded for 30 pieces of fruit for each replicate. ΔE* was calculated using Equation (2).

(2) $${\Delta E}^{*}=\sqrt{{\left({L^{*}-L^{*}}_{0}\right)}^{2}+{\left(a^{*}-a_{\;\;\;0}^{*}\right)}^{2}+{\left(b^{*}-b_{\;\;\;0}^{*}\right)}^{2}}$$

where L*_0_, a*_0_, and b*_0_ denote the initial color parameters of fresh fruit.

### 2.3. Respiration Rate and Malondialdehyde Content

Approximately 60 g of fruit was weighed and placed in a 250 mL sealed container. The accumulated CO_2_ was quantified using a Fruit and Vegetable Respiration Analyzer (3051H, Zhejiang Top Cloud-agri Technology Co., Ltd., Hangzhou, China). Respiration rate was expressed as mg CO_2_/kg·h.

Malondialdehyde (MDA) content was determined using the thiobarbituric acid (TBA) method [31]. Briefly, 0.1 g of blueberry homogenate was extracted with 5 mL of a 10% (*w*/*v*) trichloroacetic acid (TCA) solution and then centrifuged at 10,000 r/min for 10 min. Then, 2 mL of the supernatant was mixed with 2 mL of a 0.6% (*w*/*v*) TBA solution in a boiling-water bath for 20 min. Absorbance was measured at 532 nm and 600 nm. Results were expressed as μmol/g of fresh weight (FW) according to Equation (3).

(3) $$MDA\;content\;(\mu mol/g)=\frac{(A_{532}-A_{600})\times V\times 10^{6}}{\varepsilon \times l\times W}$$

where V is total reaction volume (L); *ε* is 1.55 × 10^5^ L/mol·cm, the molar extinction coefficient of the MDA–TBA complex; *l* is 1 cm, the optical path length of the cuvette; and W is sample fresh weight (g).

### 2.4. SSC, TA and Ascorbic Acid Content

Blueberry samples were homogenized and filtered to obtain the juice with which to measure the soluble solid content (SSC) using a portable digital refractometer (PAL-1, ATAGO Co., Ltd., Tokyo, Japan). Ten grams of blueberry homogenate was mixed with 50 mL of distilled water and extracted for 30 min at 90 °C. The supernatant, obtained via centrifugation at 8000 r/min, was titrated with 0.01 mol/L of NaOH to test the titratable acidity (TA) content. The result was expressed as citric acid equivalents (%). The ascorbic acid content was determined using the molybdenum-blue colorimetric method, as described by Elnenaey et al. [32]. Briefly, 1 g of blueberry fruit sample was ground with 5 mL of 0.4% (*w*/*v*) oxalic acid solution in 0.1% (*w*/*v*) EDTA. After centrifugation at 4000 r/min for 10 min, 1.0 mL supernatant was mixed with 0.1 mL of 3% metaphosphoric in 8% acetic acid solution and 0.2 mL of 5% sulfuric acid solution. Then, 0.4 mL of 5% (*w*/*v*) ammonium molybdate solution was added, and the mixture was diluted to 5 mL with distilled water. The absorbance at 705 nm was measured, and the calculation was done based on a standard curve of ascorbic acid.

### 2.5. Enzyme Activity

Blueberry tissue (0.5 g) was homogenized in 2.5 mL phosphate buffer (0.1 M, pH 6.8) in an ice bath. The homogenate was centrifuged at 10,000 r/min for 30 min at 4 °C, and the resulting supernatant was collected as the crude enzyme extract.

Peroxidase (POD) activity was determined according to the method described by Manda-Hakki et al. [33] with minor modifications. The reaction mixture consisted of guaiacol solution (3.0 mL, 20 mM), enzyme extract (0.5 mL), and H_2_O_2_ solution (200 µL, 0.5 M). The addition of H_2_O_2_ initiated the reaction, and the absorbance at 470 nm was measured every 10 s for 1 min. One unit (U) of POD activity was defined as a 0.01 increase in absorbance per min.

For polyphenol oxidase (PPO) activity, the reaction contained catechol solution (2.9 mL, 0.1 M) and enzyme extract (0.1 mL). After mixing, the absorbance was measured at 420 nm for 3 min at 30 s intervals. One unit (U) of PPO activity was defined as an increase of 0.01 in absorbance per min.

Superoxide dismutase (SOD) activity was determined using commercial assay kits (Solarbio Science & Technology Co., Ltd., Beijing, China) used according to the manufacturer’s instructions. The assay is based on the inhibition of WST-1 reduction by superoxide anions generated in the xanthine–xanthine oxidase system. The absorbance was recorded at 450 nm, and one unit of SOD activity was defined as the amount of enzyme required to cause 50% inhibition of WST-1 reduction under the assay conditions.

Catalase (CAT) activity was determined according to Carrión-Antolí et al.’s method [34] with minor modifications. The total reaction volume was 3.0 mL, consisting of 2.40 mL of 50 mM phosphate buffer (pH 7.0), 0.30 mL of 100 mM H_2_O_2_ working solution (with a final H_2_O_2_ concentration of 10 mM), and 0.30 mL of enzyme extract. The reaction was initiated by adding H_2_O_2_, and the absorbance at 240 nm was recorded every 30 s for 2 min. CAT activity was calculated from the rate of decrease in absorbance over time, and one unit (U) of CAT activity was defined as a decrease of 0.01 in absorbance at 240 nm per minute.

### 2.6. Total Phenol, Flavonoid, and Proanthocyanidin Content

A total of 1 g of blueberry sample was immersed in 30 mL of 70% methanol. The mixture was extracted in an ultrasonic bath at 50 °C for 60 min, and this was followed by centrifugation at 10,000 r/min for 10 min. The supernatant was collected for the following analysis.

Total phenol content was determined using the Folin–Ciocalteu method [35]. Briefly, 0.5 mL of extract supernatant was mixed with 2.5 mL of Folin–Ciocalteu reagent and incubated in a 45 °C water bath for 4 min. Subsequently, 2 mL of 7.5% (*w*/*v*) sodium carbonate solution was added and vortexed. The mixture was allowed to stand at room temperature for 30 min. Absorbance was measured at 750 nm, and results were expressed as mg gallic acid equivalents per 100 g FW (mg/100 g).

The total flavonoid content was determined using a modified colorimetric method [36]. A 0.5 mL aliquot of fruit extract was mixed with 0.5 mL of 5% (*w*/*v*) NaNO_2_ solution. After 6 min, 0.5 mL of 10% Al(NO_3_)_3_ (*w*/*v*) solution was added, and we waited for an additional 6 min. Then, 3 mL of 4% NaOH solution was added. We kept the reaction mixtures at room temperature for 20 min. Absorbance was recorded at 510 nm, and rutin was used as the standard.

Proanthocyanidin content was determined using a modified vanillin–HCl method [37]. In brief, 0.5 mL of extract was added to a test tube containing 3 mL of 5% (*w*/*v*) vanillin–methanol solution and 1.5 mL of concentrated HCl. The mixture was vortexed and allowed to react in the dark at room temperature for 1 h. Absorbance was measured at 500 nm, and the results were expressed as mg cyandin-3-glucoside equivalents per 100 g FW (mg/100 g).

### 2.7. Targeted Polyphenol Metabolomics Analysis

#### 2.7.1. Sample Extraction

Based on the content of total phenols, flavonoids, and proanthocyanidins, 21 d was selected as the sampling point for further polyphenol metabolomic analysis, as significant differences between the control (21d-CK) and optimal SMF pretreatment (21d-MF) group had appeared. Fresh fruit (0 D) was examined concurrently for comparison. Polyphenol extraction was conducted in accordance with the standard procedures. Briefly, blueberry samples were freeze-dried and finely ground. Approximately 30 mg of powder was extracted with pre-cooled 70% methanol containing an internal standard. The extracts were centrifuged at 12,000 r/min and filtered through a 0.22 µm PTFE membrane before analysis.

#### 2.7.2. UHPLC and Mass-Spectrometry Conditions

Ultra-high-performance liquid chromatography coupled with electrospray ion source tandem mass spectrometry (UHPLC–ESI-MS/MS) was performed to evaluate targeted polyphenol metabolomics. An Agilent 1290 Infinity UPLC system was used under the following conditions. The column used was an Agilent SB-C18 (1.8 μm, 2.1 mm × 100 mm). The mobile phase consisted of solvent A (pure water with 0.1% formic acid) and solvent B (acetonitrile with 0.1% formic acid). Sample measurements were performed using a gradient program with the following starting conditions: 95% A, 5% B. Within 9 min, a linear change to 5% A and 95% B was programmed and kept for 1 min. Subsequently, the composition was changed to 95% A and 5% B over 1.1 min and maintained for 2.9 min. The flow velocity was set as 0.35 mL per minute; the column oven temperature was 40 °C; and the injection volume was 2 μL.

An electrospray ionization (ESI) triple-quadrupole linear ion trap (QTRAP) mass spectrometer (Applied Biosystems 6500 QTRAP) was used to analyze the sample metabolites. The ESI source operation parameters were as follows: source temperature—500 °C; ion spray voltage (IS)—5500 V (positive ion mode)/−4500 V (negative ion mode); ion source gas I (GSI), gas II (GSII), and curtain gas (CUR)—50, 60, and 25 psi, respectively; and collision-activated dissociation (CAD)—high. Multiple-reaction monitoring (MRM) analysis was used to perform triple-quadrupole (QQQ) mass spectrometry. DP (de-clustering potential) and CE (collision energy) for individual MRM transitions were established with further DP and CE optimization. A specific set of MRM transitions was monitored for each period according to the metabolites eluted within this period, eliminating interference from non-target ions and making quantification more accurate and reproducible.

#### 2.7.3. Metabolite Identification and Quantification

Metabolites were identified based on their fragmentation patterns, retention times, and mass-to-charge ratio relative to a self-built MWDB database, as secondary spectral information was used to characterize the metabolites (Metware Biotechnology Co., Ltd., Wuhan, China). The quantification was accomplished via MRM using triple quadrupole (QQQ) scan mode. After metabolic chromatographic peaks were obtained, Analyst 1.6.3 software was used to process mass-spectrometry data for metabolite quantification based on the in-house Metabolite Database. A mass-spectrometry file of the sample was created using MultiQuant software (version 3.0.3), for the integration and calibration of chromatographic peaks. The area of each chromatographic peak representes the relative content (RC) of the corresponding substance.

### 2.8. Antioxidant Capacities

The extract of the total phenols was used to measure antioxidant capacity. DPPH (1,1-diphenyl-2-picrylhydrazyl) radical-scavenging activity was assayed according to Aşik et al.’s method [38] with minor modification. A 0.05 mL aliquot of blueberry extract was mixed with 3.8 mL of a freshly prepared DPPH solution (0.2 mM in 70% methanol) and vortexed. After incubation at 37 °C for 30 min in the dark, absorbance was measured at 517 nm. A solvent control consisting of 70% methanol was analyzed under the same conditions.

ABTS (2,2′-azino-bis-3-ethylbenzthiazoline-6-sulphonic acid) scavenging capacity was evaluated using a modified version of the method developed by Ashtalakshmi and Prabakaran [39]. The ABTS radical cation was produced by mixing an aqueous ABTS stock solution (7 mM) with potassium persulfate (final concentration: 2.45 mM). The reaction mixture was incubated in the dark at room temperature for 12~16 h. For the assay, 0.1 mL of the sample solution was mixed with 3.9 mL of the generated ABTS solution, vortexed vigorously, and then incubated in the dark for 2 min. Absorbance was recorded at 745 nm.

### 2.9. Statistical Analysis

Data were expressed as the mean ± standard deviation (SD). The statistical analyses were performed using one-way analysis of variance (ANOVA) based on Duncan multiple comparisons at *p* < 0.05 using SAS 9.4 (SAS Inc., Chicago, IL, USA). Potential differential metabolites were identified using Orthogonal Partial Least Squares Discriminant Analysis (OPLS-DA) with Variable Importance in Projection (VIP) scores (VIP > 1). Polyphenol compounds with a fold change (FC) value ≥ 2 or ≤0.5, VIP > 1, and an adjusted *p* < 0.05 were considered significantly differentially altered metabolites. KEGG (Kyoto Encyclopedia of Genes and Genomes) annotation of significantly differential phenolics was conducted, and results showed the critical pathways with the highest correlation with differential compounds (http://www.kegg.jp/kegg/pathway.html) (accessed on 14 October 2025). Principal component analysis (PCA), OPLS-DA, clustering heatmap, and correlation analysis were all performed using R software (version 4.3.1) (www.r-project.org).

## 3. Results

### 3.1. Influence of Different SMF Pretreatments on Storage Qualities of Blueberries

**Figure 1:**
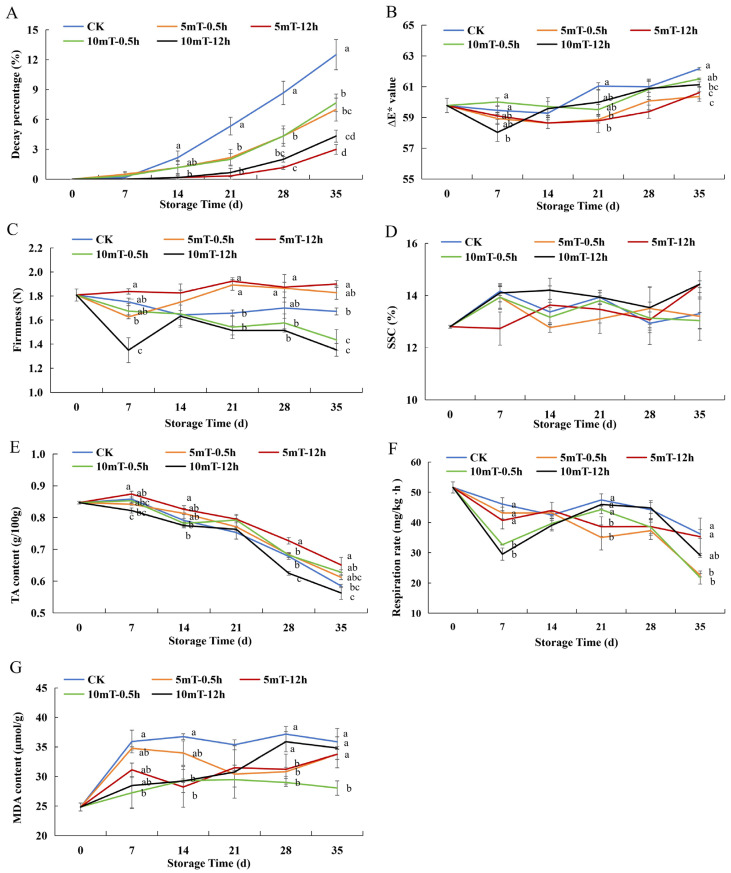
Impact of different SMF conditions on the storage qualities of blueberries during cold storage: (**A**) decay percentage; (**B**) color change; (**C**) firmness; (**D**) SSC; (**E**) TA content; (**F**) respiration rate and (**G**) MDA content. Abbreviations: ΔE*, total color difference; SSC, soluble solid content; TA, titratable acid; MDA, malondialdehyde; CK, control group. Different letters indicate significant difference (*p* < 0.05) for different groups on each storage day.

Various physiological characteristics and physical–chemical qualities were measured to optimize the SMF pretreatment parameters. As shown in Figure 1A, the decay percentage of blueberries increased gradually with the prolongation of cold storage. The control group exhibited the highest decay percentage in the period 14~35 d after treatment, whereas the SMF pretreatments effectively delayed the onset and progression of fruit decay. The 10 mT-12 h and 5 mT-12 h groups showed the most pronounced inhibition of decay (*p* < 0.05).

The ΔE* value changed progressively throughout storage, indicating a gradual change in peel color (Figure 1B). The control blueberries showed a more pronounced increase in ΔE*, whereas the 5 mT-0.5 h and 5 mT-12 h treatments significantly slowed the shift in ΔE* at 21 and 35 d (*p* < 0.05), demonstrating that the SMF pretreatments, particularly 5 mT for 12 h, delayed peel color deterioration and maintained the appearance quality of blueberries in the later stage of storage.

Blueberry firmness declined progressively during cold storage in all groups (Figure 1C). SMF pretreatment delayed the softening of fruit, with the 5 mT-0.5 h and 5 mT-12 h groups maintaining significantly higher firmness than the control in the late storage stage (5 mT-0.5 h on day 21, 5 mT-12 h on days 21 and 35) (*p* < 0.05). These findings indicate that the 5 mT-12 h SMF pretreatment effectively retarded tissue softening from the mid to late storage periods.

The SSC of blueberries initially increased slightly and then showed mild fluctuations (Figure 1D). The increment in SSC may be due to the transformation of some insoluble components into soluble sugars, such as starch, whereas the soluble substances would be partially consumed as metabolic substrates, leading to a gradual decline or fluctuation. Among the treatments, the 5 mT-12 h and 10 mT-12 h groups showed relatively higher SSC values at the end of storage but with no significance. SMF pretreatment had little impact on the content of soluble solids.

Blueberry TA content gradually declined in all samples during cold storage, reflecting the consumption of organic acids (Figure 1E). SMF pretreatment at 5 mT-12 h maintained a slightly higher content of TA on days 7~21 but showed no significance compared to the control group. Differences among treatments became significant in the later stage of cold storage. On days 28 and 35, the TA content in the 5 mT-12 h treatment group remained higher than in the control (*p* < 0.05).

All the blueberry groups exhibited a marked decrease in respiration rate during the early stage of cold storage, followed by a relatively stable phase thereafter (Figure 1F). Blueberries pretreated with 10 mT SMF maintained a lower respiration rate when stored for 7 d under cold temperatures. On days 21 and 35, the respiration rate of the 5 mT-0.5 h group was significantly reduced compared to the control (*p* < 0.05). SMF corresponding to 5 mT-12 h suppressed the respiration rate from approximately days 21 to 28, with a significant difference on day 21 (*p* < 0.05).

Malondialdehyde (MDA) is a typical marker of lipid peroxidation and membrane oxidative damage, commonly used to assess the degree of physiological deterioration in stored fruit. As storage time increased, MDA accumulated in all the groups (Figure 1G), indicating an increase in oxidative stress. Nevertheless, some SMF pretreatments significantly mitigated this trend. SMF corresponding to 5 mT-12 h significantly reduced MDA content on days 14 and 28 (*p* < 0.05). The 10 mT-0.5 h group exhibited lower MDA levels (*p* < 0.05) throughout cold storage, except on day 21. In addition, on day 28, the MDA content of the 5 mT-0.5 h group was significantly lower than that of the control and 10 mT-12 h groups (*p* < 0.05).

### 3.2. Influence of Different SMF Pretreatments on Antioxidant Enzyme Activity of Blueberries

**Figure 2:**
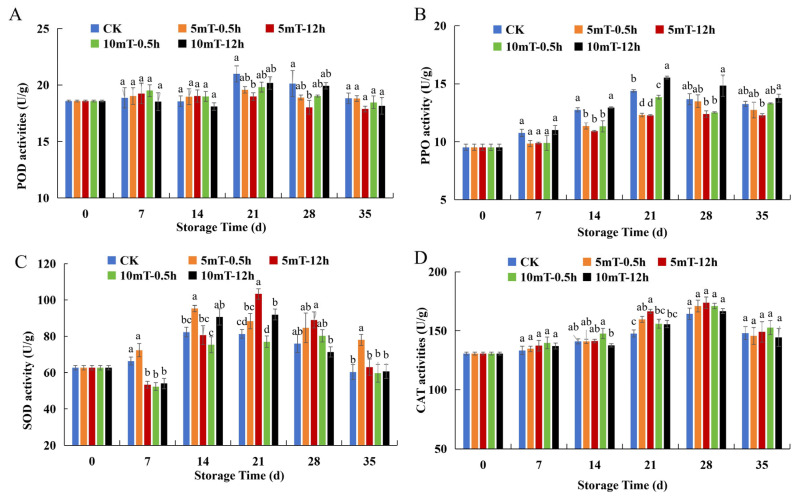
Impact of different SMF pretreatments on the enzyme activity of blueberries during cold storage: (**A**) POD activity; (**B**) PPO activity; (**C**) SOD activity; (**D**) CAT activity. Abbreviations: POD, peroxidase; PPO, polyphenol oxidase; SOD, superoxide dismutase; CAT, catalase. CK, control group. Different letters indicate significant differences (*p* < 0.05) for different groups at each storage day.

Peroxidase (POD) and polyphenol oxidase (PPO) are key enzymes closely linked to the antioxidant system, phenolic metabolism, and browning incidence [40]. The POD activity of blueberries first increased and subsequently decreased during storage (Figure 2A). Compared with the control, SMF pretreatments caused only slight changes in POD activity during storage. On days 21 and 28, some divergence among treatments became evident. POD levels in the 5 mT-12 h group were significantly lower than in the control group (*p* < 0.05). The PPO activity of the stored blueberries exhibited an increasing trend in the early–mid-storage period (7~21 d), followed by a relatively stable change (Figure 2B). Throughout the 14~21 d period, the PPO activity of several of the groups exposed to SMF treatment, especially the 5 mT-12 h group, was significantly inhibited compared to the control (*p* < 0.05). As shown in Figure 2C, SOD activity exhibited a trend of first increasing and subsequently decreasing during cold storage. In the early storage stage, 5 mT-0.5 h led to a relatively rapid increase in SOD activity, whereas 5 mT-12 h maintained the highest SOD levels from approximately days 21 to 28, with a significant difference relative to the control (*p* < 0.05). CAT activity displayed similar trends but reached a peak value on day 28 (Figure 2D). Overall, the SMF pretreatments maintained higher CAT activity during the 14~28 d period. Among the treatments, 5 mT-12 h resulted in significantly higher CAT activity on day 21 (*p* < 0.05).

### 3.3. Influence of Optimal SMF Pretreatment on Non-Enzymic Antioxidant Levels of Blueberries

**Figure 3:**
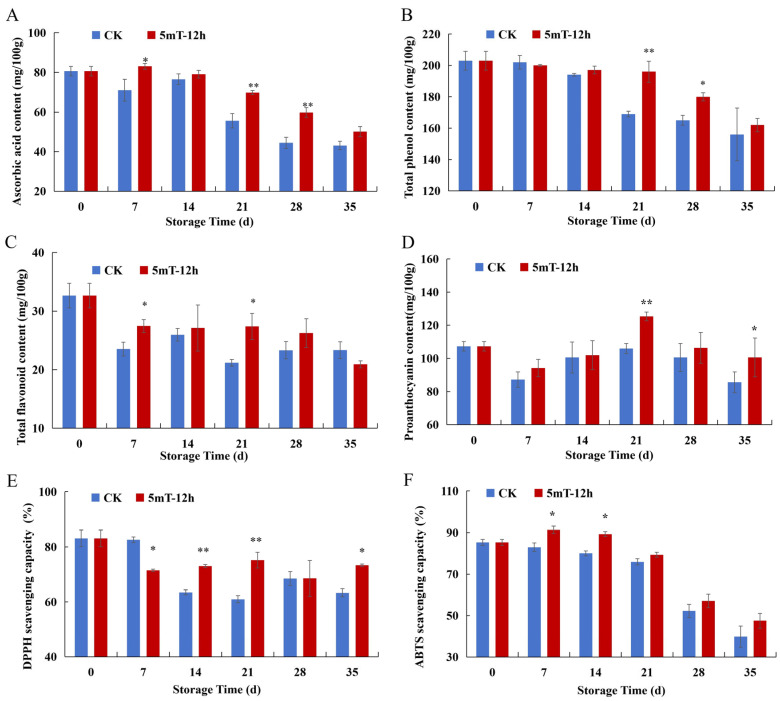
Impact of optimal SMF pretreatment on non-enzyme antioxidant levels of blueberries during cold storage: (**A**) ascorbic acid content; (**B**) total phenol content; (**C**) total flavonoid content; (**D**) proanthocyanin content; (**E**) DPPH radical-scavenging activity; (**F**) ABTS radical-scavenging activity. Abbreviations: DPPH, 1,1-diphenyl-2-picrylhydrazyl; ABTS, 2,2′-azino-bis-3-ethylbenzthiazoline-6-sulphonic acid. CK, control group. Here, 5 mT-12 h is the optimal static magnetic field condition. * and ** indicate significant difference at *p* < 0.05 and *p* < 0.01 between the optimal SMF pretreatment and control groups, respectively.

Collectively, the quality metrics and enzyme indicators demonstrate that pretreatment with a 5 mT SMF for 12 h was the optimal condition for blueberries. Subsequently, its influence on antioxidant components was investigated. Ascorbic acid (AsA) is a key antioxidant component associated with the nutritional quality and oxidative stability of fruit [41]. In this study, ascorbic acid levels decreased during cold storage, especially after 21 d. The control group exhibited significantly lower AsA content compared to the SMF-pretreated samples on days 7, 21, and 28 (Figure 3A), suggesting the SMF pretreatment mitigated the oxidative degradation of ascorbic acid.

Polyphenols are important secondary metabolites with antioxidant and antimicrobial properties contributing to postharvest fruit quality and nutrition. The total phenol content of blueberries exhibited a gradual decline in the control group (Figure 3B), reflecting oxidative consumption of phenolic constituents. No significant differences were observed in the early storage stage (0~14 d). However, the influence of SMF pretreatments became evident on 21~28 d (*p* < 0.05). SMF pretreatment effectively preserved the phenolic compounds of blueberries during long-term cold storage. The total flavonoid content generally decreased during cold storage (Figure 3C). However, the decline was retarded in the SMF-pretreated fruit. Significantly higher flavonoid content was found in the 5 mT-12 h SMF group relative to the control on days 7 and 21 (*p* < 0.05).

Proanthocyanidins, as key phenolic compounds in blueberries, have a critical influence on astringency and antioxidant capacity [42]. As shown in Figure 3D, the proanthocyanidin content in both groups decreased slightly in the initial stage of storage and then fluctuated during the mid-to-late storage period, which may be due to the dynamic changes in the synthesis, transformation, and consumption of certain compounds. SMF pretreatment had a significant influence on proanthocyanins. The proanthocyanidin content of the 5 mT-12 h group peaked on day 21 and was significantly higher than the control (*p* < 0.05). At the last stage of storage (35 d), the SMF group still maintained higher levels of proanthocyanidin (*p* < 0.05).

ABTS and DPPH radical-scavenging capacities were used to evaluate the antioxidant potential of blueberries during cold storage. Since these indices reflect the integrated antioxidant capacity of multiple antioxidants, their variation patterns might be inconsistent with some specific polyphenolics. The changes in DPPH radical-scavenging capacity are presented in Figure 3E. In the middle storage phase (14~21 d), the 5 mT-12 h treatment maintained higher levels of DPPH scavenging capacity (*p* < 0.05). At the end of storage, the 5 mT-12 h treatment still preserved higher DPPH radical-scavenging ability. As illustrated in Figure 3F, the 5 mT-12 h SMF pretreatment also maintained higher ABTS radical-scavenging capacity than the control group during days 7~14. Taken together, although some fluctuations were observed during storage, SMF pretreatment generally enhanced the antioxidant capacity of blueberries.

### 3.4. Influence of Optimal SMF Pretreatment on Polyphenol Metabolism in Blueberries

The results above demonstrate that the 5 mT-12 h SMF pretreatment significantly induced accumulation of polyphenols on day 21. Understanding how the SMF pretreatment modulated phenolic metabolism is indispensable for revealing its preservation mechanism. Targeted polyphenol metabolomic analysis was performed.

**Figure 4:**
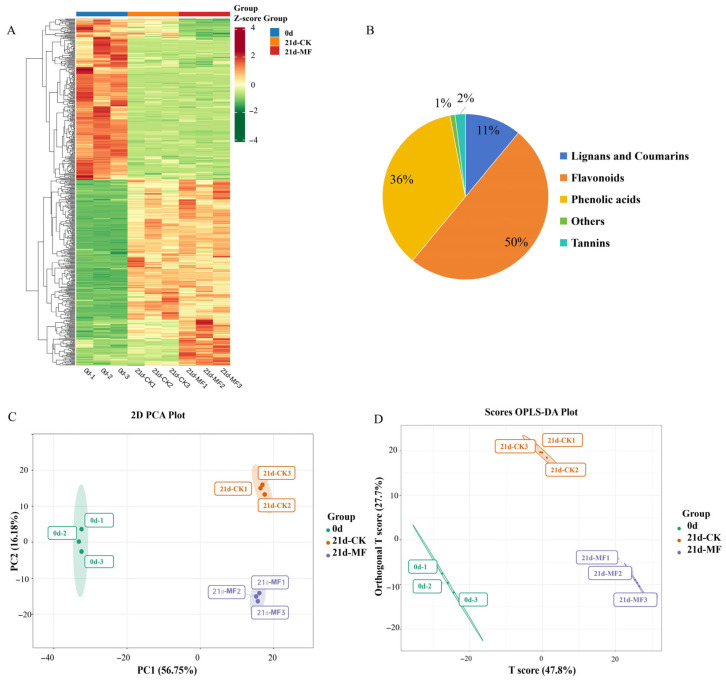
Differentially accumulated polyphenolic metabolites in blueberries: (**A**) polyphenol metabolite heatmap and hierarchical cluster analysis for different blueberries (0 d, 21d-CK, 21d-MF); (**B**) polyphenol metabolite category; (**C**) PCA plot; (**D**) OPLS-DA plot. Abbreviations**:** CK, control group; MF, static magnetic field pretreatment under optimal conditions (5 mT-12 h); 0 d and 21 d correspond to the sample at harvest and after storage for 21 d, respectively.

In total, 1058 polyphenol metabolites were detected across all samples. The hierarchical clustering heatmap in Figure 4A shows that the fresh fruit (0 d), 21d control group (21d-CK) and 21d static magnetic field group (21d-MF) were separately clustered. This indicates substantial metabolic differences among different samples. The increase in the quantity of polyphenols throughout storage was greater in the SMF pretreatment group, a finding that is in accordance with the total content of polyphenols, suggesting more phenolics were biosynthesized. As shown in Figure 4B, the identified polyphenolics were distributed across five primary categories. Flavonoids accounted for the highest proportion (50%), followed by phenolic acids (36%). Lignans and coumarins, tannins, and others accounted for 11%, 2%, and 1%, respectively. Principal component analysis (PCA) (Figure 4C) revealed that PC1 and PC2 accounted for 56.75% and 16.18% of the total variance, respectively. Samples within each treatment were closely grouped, demonstrating good replicability and metabolic consistency among replicates. The distinct separation of different samples showed an overall separation trend, suggesting differences in phenolic profiles among treatments. The OPLS-DA plot in Figure 4D shows that all the samples lie within their confidence intervals and are clearly separated, a finding consistent with the PCA result.

#### 3.4.1. Differentially Accumulated Phenolic Metabolites in Blueberries

**Figure 5:**
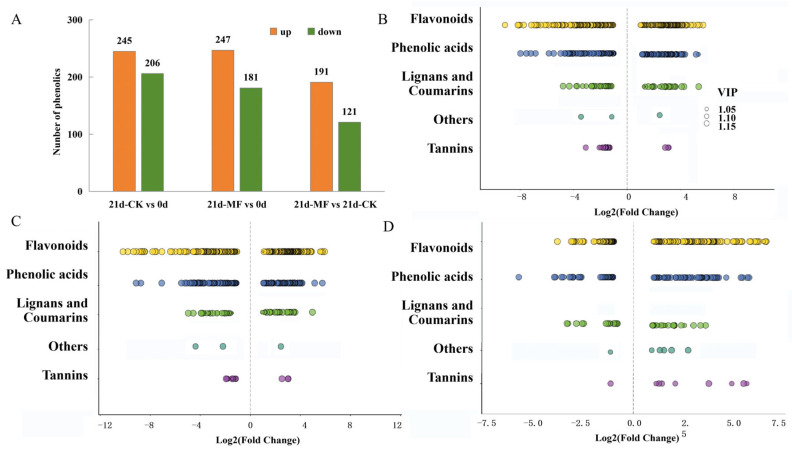
Differentially accumulated polyphenolic metabolites in blueberries: (**A**) The number of differentially accumulated phenolics (DAPs) in different groups. Orange indicates the upregulated DAPs, and green represents the downregulated ones. (**B**) Different categories of DAPs, comparing 21d-CK with 0 d, (**C**) 21d-MF with 0 d, and (**D**) 21d-MF with 21d-CK. The horizontal axis shows the Log_2_FC value, and the vertical axis indicates the different categories of phenolics. One dot represents a phenolic compound. The size of a dot reflects the VIP value. Abbreviations: CK, control group; MF, static magnetic field pretreatment under optimal conditions (5 mT-12 h); 0 d and 21 d denote the sample at harvest and after storage for 21 d, respectively.

Differentially accumulated phenolics (DAPs) were identified from the OPLS-DA model using the thresholds of a variable weight value (VIP) > 1, a fold change (FC) ≥2 or ≤0.5, and *p* < 0.05. In the control samples, 245 phenolics were significantly upregulated, and 206 were downregulated, after 21 d of cold storage (21d-CK vs. 0 d, Figure 5A). However, in the blueberries stored for 21 d after SMF pretreatment, 247 DAPs were upregulated, and 181 were downregulated (21d-MF vs. 0 d). Comparison between the groups subjected to an SMF and the control on the same storage day (21d-MF vs. 21d-CK) showed that the quantities of 191 DAPs increased, while 121 decreased. These differences in DAPs suggest that polyphenol metabolic remodeling occurred after the SMF pretreatment, with more polyphenol synthesis.

Categories of DAPs are shown in Figure 5B–D. Flavonoids and phenolic acids are the phenolic compounds that underwent the most significant changes in all three comparisons. Relative to the untreated blueberries, the differential phenolics in the SMF-pretreated samples included 168 flavonoids (104 up- and 64 downregulated) and 97 phenolic acids (58 up- and 39 downregulated), which collectively accounted for 84.9%. There were more upregulated flavonoids and phenolic acids in the SMF pretreatment group than in the control group.

#### 3.4.2. Analysis of Overall Changes in Phenolic Metabolic Pathways

**Figure 6:**
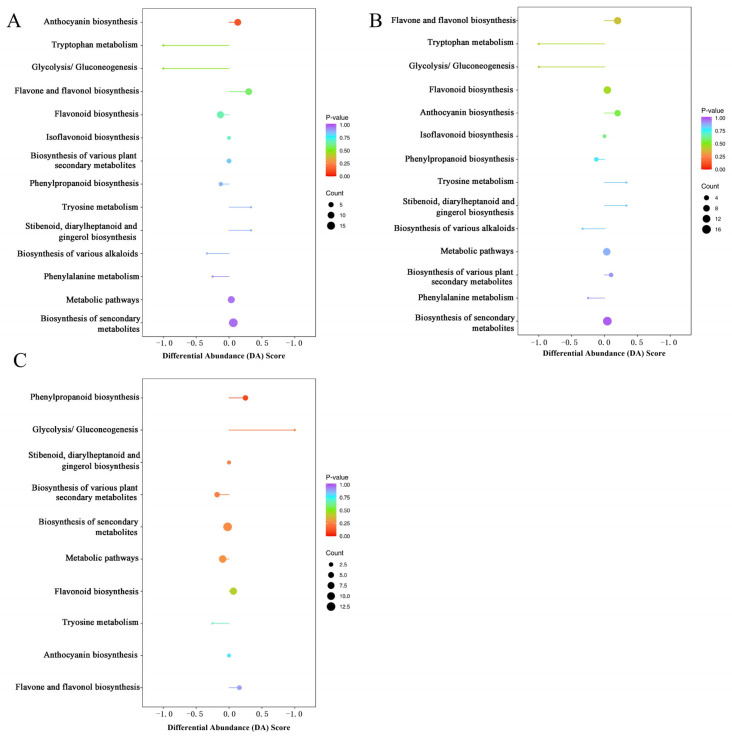
Metabolic pathway analysis of different groups: (**A**) differential abundance score (DA score) of 21d-CK vs. 0 d, (**B**) 21d-MF vs. 0 d, and (**C**) 21d-MF vs. 21d-CK. The vertical axis represents the differential pathway names (sorted by *p*-value), and the horizontal axis represents the DA score. A positive score indicates an upregulation of the expression trend of all identified metabolites in the pathway, while a negative score indicates a downregulation. The length of the line segment represents the absolute value of the DA score, and the sizes of the dots at the endpoints of the line segment represent the number of differential metabolites in the pathway. Abbreviations: CK, control group; MF, static magnetic field pretreatment under optimal condition (5 mT-12 h); 0 d, at harvest; 21 d, after cold storage for 21 d.

To construct the potential biochemical networks and cover the critical gaps in our understanding of the preservation mechanism of SMF pretreatment associated with polyphenol metabolism, the significantly altered polyphenol compounds were annotated against the KEGG database, and the resulting pathways and differential abundance scores (DA score) are illustrated in Figure 6A–C. For 21d CK vs. 0 d, 76 DAPs were annotated to 14 pathways, with biosynthesis of secondary metabolites possessing the most DAPs, followed by flavonoid biosynthesis, with 11 DAPs, and anthocyanin biosynthesis and flavone and flavonol biosynthesis, with 10 DAPs (Figure 6A). The most significantly enriched polyphenol-biosynthesis-related pathway influenced by cold storage was anthocyanin biosynthesis. Comparing 21d-MF with 0 d, 69 DAPs were annotated to 14 pathways (Figure 6B). Ten pathways annotated 47 DAPs in 21d-MF vs. 21d-CK, and phenylpropanoid biosynthesis was identified as the most significantly regulated pathway (Figure 6C). The DA Score reflected the overall changes of metabolites in specific pathways. Distinct regulation of anthocyanin and phenylpropanoid pathways was significantly induced by cold storage and SMF treatment, respectively.

#### 3.4.3. Characteristic Polyphenol Compounds’ Responses to SMF Pretreatment and Cold Storage

The significant differential polyphenols contributing to the major phenolic metabolism pathways (biosynthesis of phenylpropanoids, flavonoids, flavone and flavonols, and anthocyanins) were considered the crucial phenolics regulated by SMFs and cold duration. The relative content (RC) of these phenolics, as well as the fold change (FC) values of different comparisons, is listed in Table 1, Table 2, Table 3 and Table 4. Phenolic compounds annotated in phenylpropanoid biosynthesis were differently influenced by SMFs and cold duration (Table 1). Levels of 5-O-*p*-coumaroylquinic acid increased in both groups after cold storage, whereas 1-O-sinapoyl-D-glucose and caffeic acid levels diminished. However, some phenolic acids showed a significant difference under SMF pretreatment. Chlorogenic acid markedly accumulated after cold storage, but the SMF reduced its content 0.11-fold relative to the control. By contrast, SMF pretreatment selectively enhanced the formation of certain hydroxycinnamic acids, including caffeic acid, vanillic acid, and coumaric acid.

Within the flavonoid biosynthesis pathway, multiple compounds exhibited substantial changes (Table 2). Chrysin, taxifolin, and dihydrokaempferol all accumulated after cold storage. In addition, the levels of several flavonoid precursors decreased markedly after storage, indicating the degradation and transition of these flavonoids, including naringenin, epicatechin, catechin, phlorizin, dihydromyricetin, and 3,5,7-trihydroxyflavanone. Some flavonoids showed modifications that seemed to have been induced by the SMF. Although dihydrokaempferol and taxifolin levels increased after cold storage, their levels in the SMF-pretreated fruit were significantly lower than in the control group. The levels of multiple glycosylated derivatives, such as kaempferol-3-O-β-xylosyl-(1→2)-glucoside, dihydrokaempferol-3-O-glucoside, and isorhamnetin-3-O-sophoroside, also declined sharply in response to SMF pretreatment. In contrast to these reductions, dihydromyricetin and quercetin were the two flavonoids in this pathway that exhibited a clear SMF-induced elevation. Overall, cold storage and SMF pretreatment concurrently influence the flavonoid biosynthesis pathway, as SMF treatment exerted additional, branch-specific effects, characterized by reduced levels of taxifolin, dihydrokaempferol, and their related glycosides, whereas elevated levels of quercetin and dihydromyricetin were observed.

The levels of several kaempferol- and quercetin-derived glycosides in the flavone and flavonol biosynthesis increased markedly after cold storage (Table 3). As shown in the results, kaempferol-3-O-glucoside, kaempferol-3-O-galactoside, kaempferol-3-O-rutinoside, and kaempferol-3-O-rhamnoside-7-O-glucoside in the control blueberries all accumulated after cold storage. Similarly, quercetin-3-O-(6″-O-malonyl) glucoside and quercetin-3-O-rutinoside showed substantial storage-driven increases in abundance, as did apigenin-6-C-glucoside. Quercetin-3-O-rhamnoside, kaempferol-3-O-rhamnoside, and luteolin-7-O-rutinoside showed a significant decline during storage. Meanwhile, several flavonols exhibited clear SMF-regulated responses. Under SMF exposure, hesperetin-7-O-rutinoside and hesperetin-7-O-neohesperidoside biosynthesis increased 13.63-fold and 12.11-fold relative to 0 d, respectively. In contrast, luteolin-7-O-rutinoside levels in the SMF-pretreated blueberries decreased by 46%. These results suggest the SMF treatment exerted additional effects on several kaempferol and hesperetin downstream compounds, characterized by a general increase in the quantity of glycosylated hesperetin and a reduction in kaempferol glycosylation.

The anthocyanin biosynthesis pathway exhibited remarkable changes regulated by SMF pretreatment. Six acylated, malonylated, and glycosylated cyanidins and delphinidins showed substantial increases after cold storage (Table 4). Meanwhile, the levels of cyanidin-3-O-sambubioside, pelargonidin-3-O-rutinoside, delphinidin-3-O-(6-caffeoyl)-β-D-glucoside, and delphinidin-3-O-sambubioside decreased notably after 21 d of cold storage. Different derivatives showed various responses to cold-temperature storage. However, SMF pretreatment inhibited the changes in some anthocyanins, with the delphinidin-3-O-(6-caffeoyl)-β-D-glucoside and delphinidin-3-O-(6″-O-p-coumaroyl) glucoside content predominantly increasing in the SMF group, while the levels of several cyanidin glucosides markedly decreased. SMF pretreatment exerted additional influences on specific structural subclasses, such as coumaroylated, malonylated and caffeoylated delphinidin and cyanidin derivates.

### 3.5. Comprehensive Effect of SMF Pretreatment on Blueberry Quality and Phenolics

**Figure 7:**
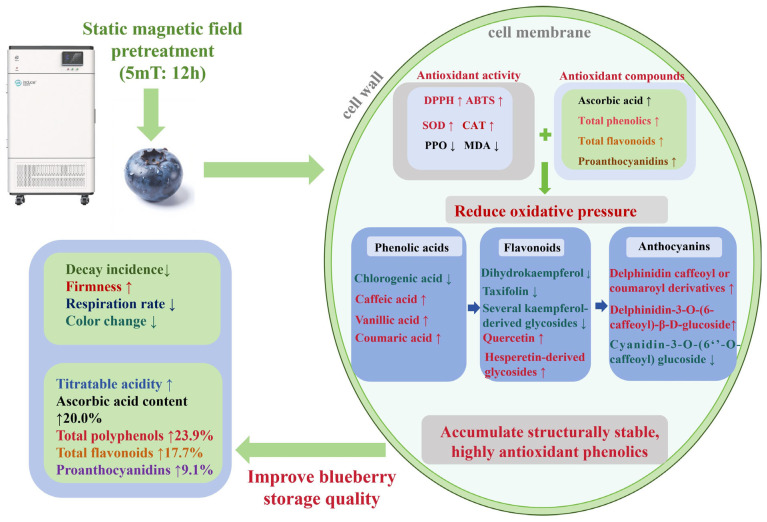
Effect of SMF pretreatment on blueberry quality and phenolics during cold storage: An upward-pointing arrow indicates the attribute is upregulated, and a downward-facing arrow means it is downregulated. Abbreviations: DPPH, 1,1-diphenyl-2-picrylhydrazyl-scavenging ability; ABTS, 2,2′-azino-bis-3-ethylbenzthiazoline-6-sulphonic acid scavenging ability; CAT, catalase; PPO, polyphenol oxidase; SOD, superoxide dismutase; MDA, malondialdehyde.

Based on the findings noted above, a schematic model (Figure 7) was established to illustrate the integrated effect of SMF pretreatment on blueberry quality and phenolic metabolites during cold storage, involving enhanced cellular antioxidant activities, increased accumulation of antioxidant compounds, and a shift in phenolic metabolism toward structurally stable and highly antioxidant phenolic derivatives.

## 4. Discussion

### 4.1. SMF Pretreatment Improves the Physiological Qualities of Blueberries During Cold Storage

Our systematic evaluation of physical and physicochemical quality indicators revealed that the optimal static magnetic field (SMF) pretreatment condition was 5 mT-12 h for blueberry cold storage. The decay, softening, and color change of blueberries were all significantly delayed by this optimal treatment. After the blueberries had been stored for 21 d, 5 mT-12 h SMF pretreatment reduced the decay percentage by 93.8% and increased firmness by 12.5% (Figure 1A,C). A similar decay-suppressing effect of SMF has also been reported in regard to cherries during cold storage [43]. In one case, the total microbial colony counts of strawberries were significantly reduced by magnetic field (MF) treatment after 5 days of storage and showed a dependence on intensity [14]. Recent research has suggested that static magnetic fields disrupt membrane permeability and interfere with Ca^2+^-related ion transport in microorganisms, thereby reducing their growth and colonization capacity [44]. Also, modification of cell surface morphology was observed during exposure to a 6 mT SMF [45]. Furthermore, the antimicrobial effect observed in one study was likely due to a disruption of electron transport chains and ATP synthesis in pathogens that was induced by a MF’s interference with microbial iron-containing proteins [46]. These mechanisms likely contributed to the markedly lower decay percentage observed in the SMF-treated blueberries during cold storage.

The greater firmness of the blueberries in the SMF group demonstrates the SMF’s positive effect on maintaining blueberry texture during long-term cold storage (Figure 1C). Similar improvements in the firmness retention of blueberries under SMF treatment have been reported by Tao et al. [47]. An SMF combined with low-temperature storage helped maintain a firmer texture in wampees, sapodillas, [18,21] and green chilies [17]. The structures and network of pectin fractions were found to be maintained by SMF treatment due to the suppression of the activity of cell-wall-degrading enzymes, which preserved the stability and integrity of the cell wall, thereby delaying softening [17,21,48]. Sun et al. [21] found that the polarity of aromatic amino acid residues in pectinase and the secondary structure of pectinase changed after magnetic field treatment.

The lower ΔE* in the 5 mT-12 h group indicated that the SMF pretreatment effectively inhibited peel-color deterioration during cold storage (Figure 1B). Similar color retention has been observed in the SMF-assisted preservation of cherries, where magnetic fields helped maintain anthocyanin stability and surface brightness [44]. The color change in fruit during storage is triggered by the enzymes PPO and POD, which catalyze pigment degradation and disrupt the anthocyanin metabolic pathway [48]. Some oxidative enzymes inducing the degradation of anthocyanin were inhibited by a magnetic field. Consequently, chromatic quality was maintained [49].

The significant suppression of the respiration rate in the SMF group suggests reduced metabolic intensity under SMF exposure (Figure 1F). Comparable respiration-lowering effects were also reported in regard to cherries and cucumbers under SMF-assisted storage. Magnetic fields dampened respiratory metabolism and retarded senescence during cold storage [15,43]. In one study, a magnetic field was reported to influence the activity and structure of respiratory-related proteases, thereby reducing enzymatic activity and respiratory metabolism [50]. This retardation of respiration was further supported by a higher titratable acid content preserved in the SMF pretreatment group (Figure 1E), serving as evidence of slower organic-acid depletion in respiration processes. Phenolic compounds also contribute to the acidity of fruit [51]. These results are in agreement with an earlier finding that SMF exposure restrains acid metabolism and maintains flavor-related acidity during postharvest storage of wampee fruit [18].

These improvements in physiological–chemical qualities indicate that SMF pretreatment, especially at 5 mT for 12 h, helped maintain a more stable metabolic state and delayed quality deterioration throughout the cold-storage period. However, a longer exposure time (12 h) for pretreatment was found to be the optimal parameter, a result that is obviously different from other reports, in which 0.5 h was found to be optimal [15,16]. This might be due to the fact that the air environment in this study was significantly different from the water media used in other studies.

### 4.2. SMF Pretreatment Elevates the Antioxidant Defense System, Preventing Blueberry Senescence

In this study, the SMF pretreatment enhanced both enzymatic and non-enzymatic antioxidant levels (Figure 2 and Figure 3). These coordinated effects strengthened the blueberries’ ability to scavenge ROS, reduced oxidative membrane damage, and ultimately delayed postharvest senescence during cold storage.

The mitigation of oxidative damage by SMF exposure was evidenced by the lower level of MDA accumulation (Figure 1G). The ability of SMFs to modulate ROS metabolism and maintain membrane stability in postharvest fruit, helping to lower oxidative damage during fruit storage, has been reported in other studies [18,47]. Besides the protective effect on the cell membrane, SMF pretreatment increased preservation of ascorbic acid, total flavonoids, total phenols, and proanthocyanidins by 20.0%, 23.9%, 17.7%, and 9.1%, respectively (Figure 3A–D). The content of total phenols and flavonoids declined after cold storage, but proanthocyanin content increased. Similar results were demonstrated in four highbush blueberry cultivars during ripening [52]. The reduction in the quantity of polyphenols and flavonoids could be a result of enzymatic oxidation, deglycosylation, and hydrolysis of phenolics, as well as free-radical consumption during storage [53]. Microbial degradation can also be attributed to the reduction in the number of phenolics. Proper postharvest storage is crucial for preserving these bioactive compounds. Higher levels of polyphenols mitigate the redundant ROS, maintaining membrane integrity, thereby delaying oxidative stress and maintaining fruit quality [29]. SMF short-term pretreatment can serve as an abiotic stress that induces the biosynthesis of resistant substances [54]. A similar improvement in the maintenance of ascorbic acid and phenolic substances under SMF exposure has been documented in regard to green bell peppers [19]. Magnetic field pretreatment (10 mT, 10 min) was reported to promote the greatest enrichment in phenolics [55]. This improved preservation of antioxidants was further supported by the observation of SMF preventing oxidant depletion by polyphenol oxidase [48].

Simultaneously, the enhancement of antioxidant defense by SMF pretreatment was aligned with the upregulation of radical-scavenging capacity (Figure 3E,F) and antioxidant enzyme activity (Figure 2C,D). Similar static-magnetic-field-induced enhancements in antioxidant capacity (DPPH and ABTS radical-scavenging activity) have been reported in regard to fresh-cut lotus root [55] and quinoa [54]. Furthermore, SOD activity in the SMF group was notably increased by 27.3% relative to the control fruit after 21 d of storage (Figure 2C), indicating greater resistance against superoxide radicals. SOD is a metalloenzyme whose major functions are to catalyze the dismutation of superoxide anion radicals into hydrogen peroxide (H_2_O_2_) and oxygen, maintain the balance of oxidative metabolism, and prevent oxidative damage. Magnetic fields have been observed to have a comparable SOD-enhancing effect on postharvest blueberries via modulating ROS metabolism [48]. In this study, blueberry CAT activity was enhanced to a higher level after 21 d of storage in the SMF pretreatment group (Figure 2D). This result is consistent with a finding observed for SMF-treated blueberries [48], cucumbers [15] and cherry tomatoes [16]. CAT catalyzes the decomposition of H_2_O_2_, and its prosthetic group of ferriporphyrins allows SMF treatment to improve its activity [15]. Meanwhile, the lower PPO activity induced by the 5 mT-12 h SMF pretreatment indicates a reduction in browning-related oxidative activity (Figure 2B). In agreement with this notion, SMF-assisted low-temperature storage also helped stabilize membrane integrity and reduce oxidative enzyme activity in *Clausena lansium* fruit [18]. Inhibition of PPO under an alternating magnetic field has similarly been reported in regard to fresh-cut apples, where it resulted in markedly delayed enzymatic browning [56]. The active sites of enzymes contain transition metal ions, which can be affected by the Lorentz force from a magnetic field, thus changing the molecular conformation and activity of enzymes [26]. Furthermore, the gene expression of proteins has been reported to be influenced by low-intensity SMFs [57], resulting in the modification of enzyme activity. It was found that *VcCAT1*, *VcSOD1*, *VcSOD2* and *VcPOD1* gene expression during fruit storage was significantly upregulated under 6 mT SMF treatment [48].

### 4.3. SMF Pretreatment Modulates the Dynamic Changes of Phenolic Metabolism

#### 4.3.1. Phenylpropanoid Biosynthesis

Phenolic compounds are mainly synthesized through the phenylpropanoid pathway and flavonoid pathway. In this study, the phenylpropanoid pathway was the most significantly enriched pathway after SMF pretreatment (Figure 6C). Phenolic acid biosynthesis begins with the conversion of phenylalanine into cinnamic acid by PAL (phenylalanine ammonia lyase). Thereafter, C4H (cinnamate 4-hydroxylase) facilitates the hydroxylation of cinnamic acid to produce *p*-coumaric acid, which is the precursor for other phenolic acids via enzymes, such as 4CL (4-coumarate-CoA ligase) [58]. Phenylpropanoid metabolism is known to be highly flexible and can be redirected toward various defense-related branches in response to stress signals [59]. Metabolomic analysis indicated that not all phenolic metabolites are uniformly augmented through SMF pretreatment; rather, they reshape carbon flux within the phenylpropanoid pathway. Compared with the control, SMF pretreatment led to a further decrease in chlorogenic acid (Table 1), likely due to its participation in antioxidant processes and oxidative degradation [60], while other the levels of downstream hydroxycinnamic acids, such as coumaric acid, caffeic acid, and vanillic acid, were significantly increased. This pattern suggests that the SMF promoted the hydrolysis or turnover of esterified caffeoylquinic acids into free phenolic acids and redirected part of the phenylpropanoid flux toward low-molecular-weight hydroxycinnamic derivatives. This is supported by the study by Wang et al. [54], in which PAL and C4H activity were enhanced after magnetic field pretreatment. Additoinally, the gene expression of *PAL* was 6.32-fold higher than the control, and *4CL3* and *4CL4* displayed the highest expression in the SMF-treated group. Furthermore, the degradation of flavonoids into simpler phenolic acids induced by SMF might be another reason. The transition of flavonoids into phenolic acids with favorable qualities under cold-temperature conditions was reported by Galani et al. [61]. Ali et al. [53] also observed compositional transitions and degradation trends, as some flavonoids broke down into simpler phenolic acids, evidenced by an increase in levels of benzoic acid and retention of *p*-coumaric acid stored at 4 °C. Related hydroxycinnamic acids have been reported to exhibit strong antioxidant and antimicrobial activity and to participate in plant defense against oxidative and microbial stress [62]. The enhanced accumulation of these free phenolic acids in the SMF treatment was consistent with the lower decay incidence (Figure 1A), MDA content (Figure 1G), and higher retention of total phenolics and antioxidant activity (Figure 3B,E,F). SMF pretreatment induced the remodeling of the phenylpropanoid pathway, thereby helping to reinforce the non-enzymatic antioxidant substances.

#### 4.3.2. Flavonoid Biosynthesis

The synthesis of flavonoid compounds is mainly regulated by the activity of enzymes such as CHS (chalcone synthase) and CHI (chalcone isomerase) [63]. Magnetic fields’ ability to promote the biosynthesis of flavonoids can be attributed to upregulation of gene expression and enzymatic activity. Magnetic field treatment (10 mT, 10 min) significantly elevated CHS and CHI activity by 21.83% and 17.32%, respectively, and expression of the *CHI1* gene increased 16.31-fold relative to the control [54]. The cyclization of naringenin chalcone was strengthened, thus facilitating substrate influx into the flavonoid biosynthetic pathway.

Many intermediates of flavonoid biosynthesis have been shown to be primarily dependent on the duration of cold temperatures (Table 2). SMF pretreatment also changed several specific flavonoid branches. In particular, the SMF group exhibited lower levels of dihydrokaempferol, taxifolin and several kaempferol-derived glycosides than the control after 21 d of storage, whereas the levels of dihydromyricetin and quercetin significantly increased. Quercetin has also been found to be more stable and better maintained under cold temperatures as opposed to room temperature [64]. This pattern implies that SMF pretreatment enhanced the entire flavonoid pathway as well as shifted carbon allocation from the kaempferol branch toward the quercetin branch. Phenylpropanoid-flavonoid networks are organized in such a way that changes in upstream flux or branch-specific enzymes can preferentially direct carbon skeletons into distinct end products with different bioactivities [61]. Quercetin-type flavonoids generally exhibit stronger radical scavenging capacity than many precursor flavanones and dihydroflavonols [65]. SMF pretreatment also affected the composition of flavone/flavonols (Table 3). The levels of most kaempferol glycosides annotated in this pathway increased in the control group after cold storage, whereas SMF pretreatment remarkedly inhibited their synthesis. Interestingly, quercetin-based glycosides (e.g., quercetin-3-O-rutinoside and quercetin-3-O-sophoroside), acacetin, luteolin-7-O-rutinoside, and hesperetin glycosides were markedly upregulated by SMF pretreatment during storage. Hesperetin, existing in the form of its own glycosides, has multiple biological activities, including antioxidant properties, and serves to reduce DNA damage, abnormal cell morphology, and oxidative stress [66].

The SMF-induced increase in the levels of quercetin, dihydromyricetin, and hesperetin-glycoside-type flavonoids, together with the maintenance of total flavonoid content (Figure 3C) and the enhancement of DPPH and ABTS radical-scavenging capacity (Figure 3E,F), supports the view that SMF-guided flavonoid biosynthesis can lead to more potent antioxidant end products, which cooperate with SOD, CAT and POD activity to alleviate oxidative damage.

#### 4.3.3. Anthocyanin Biosynthesis

As anthocyanin derivates, cyanidin- and delphinidin-type glycosides, especially the *p*-coumaroyl and malonyl groups, substantially increased in number after cold storage (Table 4). Different anthocyanin derivatives showed various responses to cold duration and SMF pretreatment. Delphinidin-3-O-(6-caffeoyl)-β-D-glucoside and cyanidin-3-O-sambubioside levels decreased during cold storage, but cyanidin-3-O-(6″-O-caffeoyl) glucoside and cyanidin-3-O-(6″-O-p-coumaroyl) glucoside levels rose. Importantly, caffeoyl and coumaroyl-acylated delphinidin accumulated to higher levels in the SMF treatment group after storage; delphinidin-3-O-(6-caffeoyl)-β-D-glucoside levels increased by 17.02-fold relative to the control (Table 4). These results imply that the SMF selectively favored specific acylated anthocyanins. Delphinids can neutralize free radicals, protect mitochondrial membranes, and ensure stable energy production in a concentration-dependent manner [67]. Acylation and polyacylation of anthocyanins are known to increase pigment stability, enhance color retention, and improve resistance to pH, light, and temperature stress [68]. Acylated anthocyanins function as more stable colorants and bioactive compounds, with beneficial effects on oxidative and inflammatory processes [69,70]. The SMF pretreatment’s preference for caffeoyl-acylated and highly glycosylated delphinidin forms in blueberries is therefore consistent with the better maintenance of peel color with lower ΔE* values (Figure 1B) and stronger radical-scavenging capacity observed during cold storage (Figure 3E,F). Moreover, acyltransferases responsible for polyacylated anthocyanin formation have been implicated in producing structurally robust pigments under stress conditions [71]. SMF pretreatment promoted the accumulation of structurally stable anthocyanins, thereby contributing to both the maintenance of visual color quality and antioxidant defense.

**Figure 8:**
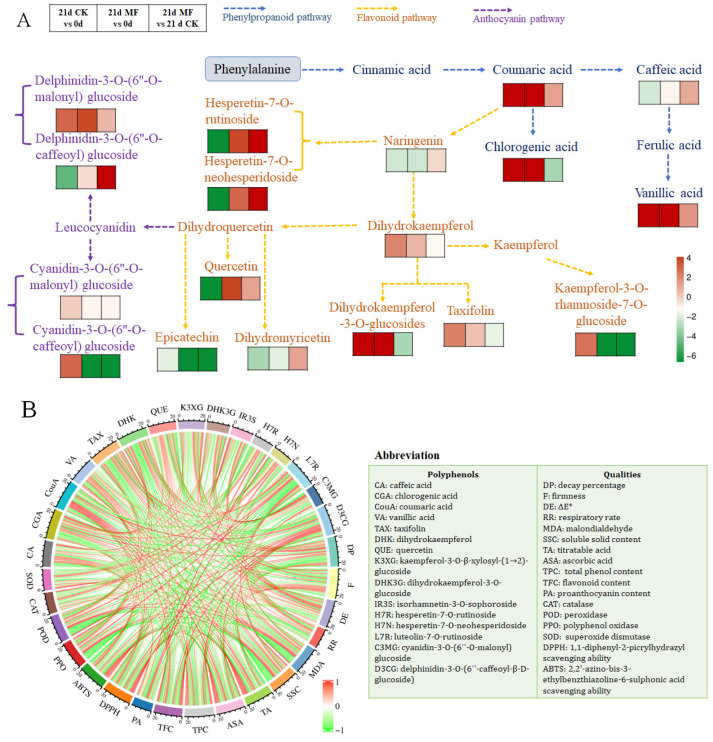
Characteristic phenolic metabolism framework and integrated analysis regarding blueberry storage qualities and antioxidant indicators: (**A**) Effects of regulation of cold storage and static magnetic field (SMF) pretreatment on specific phenolic metabolism networks. The symbols under each compound represent the regulation effects according to the Log_2_(fold change) value in the comparision of 21d-CK vs. 0d, 21d-MF vs. 0d, and 21d-MF vs. 21d-CK, respectively. Red color indicates up-regulation and green indicates down-regulation. The depth of color indicates the Log_2_(FC) value. Compounds in blue, orange, and purple mean they are in phenylpropanoid, flavonoid, and anthocyanin pathway, respectively. (**B**) Circle correlation pearson analysis of blueberry quality, antioxidant capacities, enzyme activity and key polyphenolic compounds. Green line means negative correlation, and red line indicates positive correlation, with the thickness of the line representing the correlation coefficient. Abbreviations: CK, control group; MF, static magnetic field pretreatment; 0 d, at harvest; 21 d, after cold storage for 21 d.

A regulatory flowchart of the effects of cold storage and SMF pretreatment on some characteristic phenolics annotated in the key pathways is shown in Figure 8A. Cinnamic-acid-type phenol acids, such as coumaric acid, caffeic acid, and vanillic acid, were significantly upregulated by SMF pretreatment. Levels of quercetin and dihydromyricetin, as important intermediate substances in the flavonoid pathway, were also elevated by SMF pretreatment. Hesperetin glycoside-derivates significantly accumulated in the SMF pretreatment group, in addition to delphinidin-3-O-(6″-O-caffeoyl) glucoside in the anthocyanin pathway, whereas the dihydrokaempferol-to-taxifolin pathways that were promoted by cold storage were significantly inhibited by SMF pretreatment. We have managed to concisely and visually display the effects of SMF pretreatment on crucial phenolic biosynthesis in blueberries during cold storage.

### 4.4. Integrated Influences of SMF on Fruit Quality, Phenolic Metabolism and Antioxidant System

Furthermore, the reconfiguration of phenolic metabolism induced by SMF pretreatment should be interpreted in conjunction with changes in fruit quality, as well as enzymatic and non-enzymatic antioxidant systems. As shown in the integrated correlation heatmap (Figure 8B), several phenolic metabolites that accumulated due to the SMF, e.g., caffeic acid (CA), coumaric acid (CouA), quercetin (QUE), delphinidin-3-O-(6-caffeoyl)-β-D-glucoside (D3CG), and hesperetin-7-O-rutinoside (H7R), exhibited significant positive relationships with fruit firmness, DPPH and ABTS antioxidant capacities, and SOD and CAT activity. The results reveal the significant contribution of these phenolic compounds to blueberry antioxidant potential and deterioration control. Phenolic content and antioxidant activity generally show a positive correlation [60,72]. A synthetic effect on enrichment of phenolics and improvement of antioxidant levels in blueberries was obtained under SMF pretreatment. The same mechanism of action was reported in regard to SMF-pretreated quinoa [54].

Meanwhile, these phenolics were negatively correlated with decay percentage (DP), ΔE*, and MDA content. Antioxidant substances can scavenge accumulated ROS and maintain the integrity of the cell membrane, thereby delaying postharvest senescence [73]. Phenolics have been considered to be a barrier against the conversion of unsaturated fatty acids into saturated fatty acids, which ensures higher membrane stability and retards MDA production [29]. The enrichment of hydroxycinnamic acids, quercetin, dihydromyricetin, hesperetin-type flavonoids, and caffeoyl-acylated delphinidins induced by SMF pretreatment observed here likely provided additional structural and antioxidant support to the cell membrane, thereby completing the enzymatic antioxidant system.

Fruit firmness is a crucial parameter that can reflect the progress of senescence during storage [74]. Blueberry firmness has been found to have a significantly positive correlation with proanthocyanins, ascorbic acid and antioxidant activity. In the present study, SMF pretreatment significantly delayed blueberry softening, aligning with higher contents of proanthocyanidins and total phenols. Previous studies have reported that storage conditions increase pigment accumulation, an effect sometimes associated with better textural maintenance at appropriate temperatures [75]. Magnetic-field-assisted low-temperature storage inhibits cell-wall-degrading enzymes and reduces pectic polysaccharide breakdown, thereby preserving cell wall integrity [18,47]. The lignans more upregulated by SMF pretreatment may also have played a role in strengthening cell wall structure (Figure 5D).

Collectively, these findings suggest that SMF pretreatment reprograms phenolic metabolism toward structurally stable and highly antioxidant phenolic species, which, in conjunction with the enhanced enzymatic defenses, strengthen blueberries’ ability to resist oxidative stress and decay during cold storage. Nevertheless, this study still has some limitations, especially the lack of SMF exposure duration parameters. Future studies should optimize the key operational parameters of the SMF process, including magnetic field strength and exposure time, so as to better evaluate its feasibility and practical potential for reducing postharvest losses. Meanwhile, the quantification of the absolute levels of phenolic compounds and the revelation of the molecular basis underlying SMF-mediated metabolic regulation require further investigation.

## 5. Conclusions

In conclusion, static magnetic field (SMF) pretreatment at 5 mT for 12 h effectively alleviated oxidative deterioration in blueberries and maintained their quality over 35 d of refrigerated storage. This treatment preserved higher concentrations of ascorbic acid, total phenols, flavonoids, and anthocyanidins while enhancing DPPH and ABTS radical-scavenging capacities as well as SOD and CAT activity. In addition, SMF pretreatment markedly regulated phenolic biosynthesis and promoted the accumulation of phenolic compounds with more stable structures and higher bioactive potential, such as coumaric acid, caffeic acid, vanillic acid, quercetin, hesperetin glycosides, and caffeoyl delphinidin derivatives. These coordinated changes in phenolic metabolism and antioxidant defense collectively enhanced blueberries’ resistance to oxidative stress during cold storage. This study is of great significance for revealing the preservation mechanism of magnetic-field-assisted technology and supporting its practical application to reduce postharvest losses of fruits and vegetables.

## Figures and Tables

**Table 1:** The characteristic phenolics in phenylpropanoid biosynthesis in different blueberry groups.

	Name (Formula)	M/Z (Da)	0 d	21d-CK		21d-MF		
			RC ×10^5^	RC ×10^5^	FC vs. 0 d	RC ×10^5^	FC vs. 0 d	FC vs. 21d-CK
1	5-O-*p*-Coumaroylquinic acid (C_16_H_18_O_8_)	338.31	1.92 ± 0.05	6.17 ± 0.69	3.22 *	6.18 ± 0.92	3.22 *	1.00
2	1-O-Sinapoyl-D-glucose (C_17_H_22_O_10_)	386.35	18.46 ± 1.38	4.41 ± 0.39	0.24 *	3.66 ± 0.27	0.20 *	0.83
3	Caffeic acid (C_9_H_8_O_4_)	180.16	23.58 ± 2.69	5.99 ± 0.16	0.22 *	10.67 ± 0.53	0.45 *	2.04 *
4	Chlorogenic acid (C_16_H_18_O_9_)	354.31	−	108.99 ± 7.35	+	12.32 ± 0.02	+	0.11 *
5	Coumaric acid (C_9_H_8_O_3_)	164.16	−	1.68 ± 0.35	+	4.12 ± 0.27	+	2.45 *
6	Vanillic acid (C_8_H_8_O_4_)	168.15	−	0.23 ± 0.10	+	0.68 ± 0.02	+	2.92 *

Note: Here, 0 d and 21 d represent fresh fruit and cold storage for 21 d. CK, control; MF, static magnetic pretreatment at 5 mT for 12 h. RC is the mean value of relative content (*n* = 3). FC represents the fold-change value relative to different samples. − means this metabolite was not detected or the FC is 0. + means the FC is infinite. * indicates significant modulation based on FC ≥ 2 or ≤0.5 and VIP > 1.

**Table 2:** The characteristic phenolics in flavonoid biosynthesis in different blueberry groups.

	Name (Formula)	M/Z (Da)	0 d	21d-CK		21d-MF		
			RC ×10^5^	RC ×10^5^	FC vs. 0 d	RC ×10^5^	FC vs. 0 d	FC vs. 21d-CK
1	Chrysin (C_15_H_10_O_4_)	254.24	0.18 ± 0.07	0.47 ± 0.04	2.63 *	0.54 ± 0.04	3.05 *	1.16
2	Naringenin (C_15_H_12_O_5_)	272.25	3.97 ± 0.74	0.85 ± 0.08	0.21 *	0.72 ± 0.02	0.18 *	0.84
3	Epicatechin (C_15_H_14_O_6_)	290.27	20.28 ± 4.30	5.52 ± 0.64	0.27 *	−	−	−
4	Phlorizin (C_21_H_24_O_10_)	436.40	25.40 ± 1.10	4.53 ± 1.43	0.18 *	3.14 ± 0.54	0.12 *	0.69
5	Catechin (C_15_H_14_O_6_)	290.27	82.84 ± 1.13	39.96 ± 2.96	0.48 *	−	−	−
6	Limocitrin malonyl glucoside (C_25_H_24_O_15_)	564.45	3.95 ± 0.25	69.77 ± 4.66	17.65 *	−	−	−
7	3,5,7-Trihydroxy flavanone (C_15_H_12_O_5_)	272.25	7.07 ± 0.26	1.37 ± 0.07	0.19 *	1.14 ± 0.04	0.16 *	0.83
8	Dihydromyricetin (C_15_H_12_O_8_)	320.25	19.15 ± 0.15	2.31 ± 0.29	0.12 *	5.33 ± 0.64	0.28 *	2.31 *
9	Taxifolin (C_15_H_12_O_7_)	304.25	0.71 ± 0.03	3.24 ± 0.16	4.58 *	0.97 ± 0.18	1.37	0.30 *
10	Dihydrokaempferol (C_15_H_12_O_6_)	228.25	0.63 ± 0.03	2.64 ± 0.58	4.21 *	1.06 ± 0.40	1.69	0.40 *
11	Pinocembrin (C_15_H_12_O_4_)	256.25	0.18 ± 0.02	−	−	0.45 ± 0.08	2.47 *	+
12	2′,4′,6′-Trihydroxychalcone (C_15_H_12_O_4_)	256.25	0.15 ± 0.04	−	−	0.44 ± 0.06	2.87 *	+
13	Phloretin (C_15_H_14_O_5_)	274.27	0.24 ± 0.05	−	−	0.08 ± 0.04	0.34 *	+
14	Quercetin (C_15_H_10_O_7_)	302.24	−	5.65 ± 0.54	+	13.39 ± 0.52	+	2.37 *
15	Kaempferol-3-O-β-xylosyl-(1→2)-glucoside (C_26_H_28_O_15_)	580.49	−	0.43 ± 0.09	+	0.05 ± 0.00	+	0.11 *
16	Dihydrokaempferol-3-O-glucoside (C_21_H_22_O_11_)	450.39	−	6.14 ± 0.50	+	0.73 ± 0.00	+	0.12 *
17	Isorhamnetin-3-O-sophoroside (C_28_H_32_O_16_)	624.54	−	0.36 ± 0.07	+	0.04 ± 0.00	+	0.12 *

Note: Here, 0 d and 21 d represent fresh fruit and cold storage for 21 d. CK, control; MF, static magnetic field pretreatment at 5 mT for 12 h. RC is the mean value of relative content of compound (n = 3). FC represents the fold-change value relative to different samples. − means this metabolite was not detected or the FC is 0. + means the FC is infinite. * indicates significant modulation based on FC ≥ 2 or ≤0.5 and VIP > 1.

**Table 3:** The characteristic phenolics in flavone and flavonol biosynthesis in different blueberry groups.

	Name (Formula)	M/Z (Da)	0 d	21d-CK		21d-MF		
			RC ×10^5^	RC ×10^5^	FC vs. 0 d	RC ×10^5^	FC vs. 0 d	FC vs. 21d-CK
1	Kaempferol-3-O-rhamnoside-7-O-glucoside (C_27_H_30_O_15_)	594.52	27.82 ± 1.37	171.65 ± 13.81	6.17 *	−	−	−
2	Chrysoeriol (C_16_H_12_O_6_)	300.26	0.19 ± 0.06	0.42 ± 0.05	2.15 *	0.61 ± 0.18	3.17 *	1.47
3	Kaempferol-3-O-glucoside (C_21_H_20_O_11_)	448.38	23.93 ± 3.24	53.21 ± 5.84	2.22 *	−	−	−
4	Kaempferol-3-O-galactoside (C_21_H_20_O_11_)	448.38	22.34 ± 3.30	50.40 ± 8.91	2.26 *	−	−	−
5	Kaempferol-3-O-rutinoside (C_27_H_30_O_15_)	594.52	1.20 ± 0.04	6.06 ± 0.18	5.03 *	3.86 ± 0.35	3.20 *	0.64
6	Quercetin-3-O-(6″-O-malonyl) glucoside (C_24_H_22_O_15_)	550.43	7.35 ± 0.15	105.02 ± 2.23	14.28 *	71.90 ± 1.28	9.78 *	0.68
7	Quercetin-3-O-rutinoside (C_27_H_30_O_16_)	610.52	110.96 ± 3.40	373.08 ± 9.43	3.36 *	380.88 ± 9.36	3.43 *	1.02
8	Quercetin-3-O-rhamnoside (C_21_H_20_O_11_)	448.38	377.48 ± 24.55	1.76 ± 0.43	0.00 *	0.68 ± 0.29	0.00 *	0.39 *
9	Kaempferol-3-O-rhamnoside (C_21_H_20_O_10_)	432.38	43.98 ± 1.00	0.38 ± 0.17	0.01 *	0.13 ± 0.05	0.00 *	0.33 *
10	Apigenin-6-C-glucoside (C_21_H_20_O_10_)	432.38	0.03 ± 0.00	0.27 ± 0.21	9.14 *	0.35 ± 0.17	11.84 *	1.29
11	Quercetin-3-O-sophoroside (C_27_H_30_O_16_)	610.52	3.83 ± 0.39	−	−	15.25 ± 1.39	3.98 *	+
12	Acacetin (C_16_H_12_O_5_)	284.26	0.28 ± 0.04	−	−	1.25 ± 0.07	4.54 *	+
13	Hesperetin-7-O-rutinoside (C_28_H_34_O_15_)	610.56	0.21 ± 0.03	−	−	2.88 ± 0.63	13.63 *	+
14	Hesperetin-7-O-neohesperidoside (C_28_H_34_O_15_)	610.56	1.54 ± 0.16	−	−	18.64 ± 2.70	12.11 *	+
15	Luteolin-7-O-rutinoside (C_27_H_30_O_16_)	610.52	62.87 ± 7.13	−	−	28.68 ± 2.36	0.46 *	+

Note: Here, 0 d and 21 d represent fresh fruit and cold storage for 21 d. CK, control; MF, static magnetic pretreatment at 5 mT for 12 h. RC is the mean value of relative content (n = 3). FC represents the fold-change value relative to different samples. − means this metabolite was not detected or the FC is 0. + means the FC is infinite. * indicates significant modulation based on FC ≥ 2 or ≤0.5 and VIP > 1.

**Table 4:** The characteristic phenolics in anthocyanin biosynthesis in different blueberry groups.

	Name (Formula)	M/Z (Da)	0 d	21d-CK		21d-MF		
			RC ×10^5^	RC ×10^5^	FC vs. 0 d	RC ×10^5^	FC vs. 0 d	FC vs. 21d-CK
1	Delphinidin-3-O-sambubioside (C_26_H_29_O_16_)	597.50	3.40 ± 0.20	1.66 ± 0.36	0.49 *	−	−	−
2	Cyanidin-3-O-(6″-O-p-coumaroyl) glucoside (C_30_H_27_O_14_)	595.53	20.56 ± 1.409	155.86 ± 10.69	7.58 *	201.60 ± 58.36	9.81 *	1.29
3	Cyanidin-3-O-rutinoside (C_27_H_31_O_15_)	595.53	2.16 ± 0.26	7.57 ± 0.15	3.51 *	10.30 ± 0.38	4.78 *	1.36
4	Pelargonidin-3-O-rutinoside (C_27_H_31_O_14_)	579.53	7.78 ± 0.57	3.12 ± 0.18	0.40 *	−	−	−
5	Delphinidin-3-O-(6″-O-p-coumaroyl) glucoside (C_30_H_27_O_15_)	611.53	2.95 ± 1.20	8.88 ± 0.21	3.01 *	28.91 ± 4.24	9.81 *	3.25 *
6	Delphinidin-3-O-(6″-O-malonyl) glucoside (C_24_H_23_O_16_)	567.48	0.31 ± 0.02	2.41 ± 0.11	7.71 *	3.99 ± 0.93	12.76 *	1.66
7	Cyanidin-3-O-sambubioside (C_26_H_29_O_15_)	581.50	3.71 ± 0.15	0.70 ± 0.11	0.19 *	0.93 ± 0.25	0.25 *	1.33
8	Cyanidin-3-O-(6″-O-malonyl) glucoside (C_24_H_23_O_15_)	551.48	20.55 ± 1.39	21.40 ± 1.91	1.04	9.65 ± 1.31	0.47 *	0.45 *
9	Cyanidin-3-O-(6″-O-caffeoyl) glucoside (C_30_H_27_O_15_)	611.53	0.25 ± 0.11	1.887 ± 0.454	7.55 *	−	−	−
10	Delphinidin-3-O-(6-caffeoyl)-β-D-glucoside (C_30_H_27_O_16_)	627.52	0.86 ± 0.38	0.038 ± 0.000	0.04 *	0.66 ± 0.15	0.76	17.02 *
11	Cyanidin-3-O-(2″-O-glucosyl) glucoside (C_27_H_31_O_16_)	611.53	0.42 ± 0.16	1.13 ± 0.18	2.72 *	−	−	−

Note: Here, 0 d and 21 d represent fresh fruit and cold storage for 21 d. CK, control; MF, static magnetic pretreatment at 5 mT for 12 h. RC is the mean value of relative content (n = 3). FC represents the fold-change value relative to different samples. − means this metabolite was not detected or the FC is 0. * indicates significant modulation based on FC ≥ 2 or ≤0.5 and VIP > 1.

## Data Availability

The original contributions presented in this study are included in the article. Further inquiries can be directed to the corresponding authors.

## Abbreviations

The following abbreviations are used in this manuscript:

ABTS	2,2′-azino-bis(3-ethylbenzothiazoline-6-sulfonic acid)
ANOVA	Analysis of variance
AsA	Ascorbic acid
CAD	Collision-activated dissociation
CAT	Catalase
CE	Collision energy
CK	Control
DA	Differential abundance
DAPs	Differentially accumulated polyphenols
DP	Declustering potential
DPPH	2,2-diphenyl-1-picrylhydrazyl
ESI	Electrospray ionization
FC	Fold change
KEGG	Kyoto Encyclopedia of Genes and Genomes
MDA	Malondialdehyde
MF	Magnetic field
MRM	Multiple reaction monitoring
OPLS-DA	Orthogonal partial least squares discriminant analysis
PCA	Principal component analysis
POD	Peroxidase
PPO	Polyphenol oxidase
QQQ	Triple quadrupole
QTRAP	Triple-quadrupole linear ion trap
RC	Relative content
RH	Relative humidity
ROS	Reactive oxygen species
SD	Standard deviation
SMF	Static magnetic field
SOD	Superoxide dismutase
SSC	Soluble solids content
TA	Titratable acidity
TBA	Thiobarbituric acid
TCA	Trichloroacetic acid
UHPLC	Ultra-high-performance liquid chromatography
VIP	Variable importance in projection
